# Subtype-specific transcriptional regulators in breast tumors subjected to genetic and epigenetic alterations

**DOI:** 10.1093/bioinformatics/btz709

**Published:** 2019-09-16

**Authors:** Qian Zhu, Xavier Tekpli, Olga G Troyanskaya, Vessela N Kristensen

**Affiliations:** 1 Department of Biostatistics and Computational Biology, Dana-Farber Cancer Institute, Boston, MA 02215, USA; 2 Department of Genetics, Institute for Cancer Research, Oslo University Hospital, Radiumhospitalet, Oslo, Norway; 3 Division of Medicine, Department of Clinical Molecular Biology (EpiGen), Akershus University Hospital, Lørenskog, Norway; 4 Department of Computer Science, USA; 5 Lewis-Siegler Institute of Integrative Genomics, Princeton University, Princeton, NJ 08540, USA; 6 Simons Foundation, Flatiron Institute, New York, NY 10010, USA; 7 Institute for Clinical Medicine, University of Oslo, Oslo, Norway

## Abstract

**Motivation:**

Breast cancer consists of multiple distinct tumor subtypes, and results from epigenetic and genetic aberrations that give rise to distinct transcriptional profiles. Despite previous efforts to understand transcriptional deregulation through transcription factor networks, the transcriptional mechanisms leading to subtypes of the disease remain poorly understood.

**Results:**

We used a sophisticated computational search of thousands of expression datasets to define extended signatures of distinct breast cancer subtypes. Using ENCODE ChIP-seq data of surrogate cell lines and motif analysis we observed that these subtypes are determined by a distinct repertoire of lineage-specific transcription factors. Furthermore, specific pattern and abundance of copy number and DNA methylation changes at these TFs and targets, compared to other genes and to normal cells were observed. Overall, distinct transcriptional profiles are linked to genetic and epigenetic alterations at lineage-specific transcriptional regulators in breast cancer subtypes.

**Availability and implementation:**

The analysis code and data are deposited at https://bitbucket.org/qzhu/breast.cancer.tf/.

**Supplementary information:**

Supplementary data are available at *Bioinformatics* online.

## 1 Introduction

Tumorigenesis in breast cancer is thought to be the result of a combination of somatic genetic events including copy number alterations (CNA), point mutations and epigenetic alterations such as DNA methylation (DNAme). In contrast to normal tissue development, in tumors somatic mutations accumulate at various points of the differentiation process, making normal cells acquire properties of stem cells that turn them into cancer cells. Despite the extensive generation of molecular data, how these mutations and aberrations specifically affect transcription factors and their targets is not well understood.

Breast cancer is a heterogeneous disease comprised of several molecular subtypes: luminal A, luminal B, Her2, basal-like and normal-like (Hu *et al.*, 2006; Perou *et al.*, 2000; Sørlie and Perou, 2001). Of these, luminal A and basal-like (hitherto referred to as basal) are the most extreme in terms of spanning the prognosis and treatment decision spectrum, with luminal A being ‘best’ prognosis and basal being the worst. Aberrations in breast cancer are manifested in a subtype-specific manner, and distinct biological processes are uniquely perturbed in these subtypes (Curtis *et al.*, 2012; Nik-Zainal *et al.*, 2012). Previous efforts have identified mutational events at an unprecedented resolution and scale, linking those to the breast cancer subtypes (Cancer and Atlas, 2012). Because of the distinct expression profiles, it is thought that the development of cancer involves the combinatorial effects of transcription factors expressed in a coordinated manner. Together they evolve downstream of germ-line and somatic genetic events and subtype-specific regulators can further give rise to the subtype-specific co-expression of downstream target genes.

To study the regulatory effect on transcription, various TF regulatory networks have been constructed from ChIP-seq and ChIP-ChIP data, including a general TF network created by the ENCODE project (Dunham *et al.*, 2012; Gerstein *et al.*, 2012; Kundaje *et al.*, 2015), and for breast—a nuclear receptor TF network focused on MCF7 cells (Kittler *et al.*, 2013). Computational efforts have identified master regulators from differentially expressed and coexpressed genes (Janky *et al.*, 2014), or from motifs overrepresented in promoter regions (Joshi *et al.*, 2012; Tongbai *et al.*, 2008). Some identified transcriptional differences in normal breast development through histone marks (Pellacani *et al.*, 2016). These previous analyses described a general disease network and have not been breast cancer subtype-specific. Given that subtypes are strongly linked to clinical outcome, subtype-specific regulatory networks need to be identified and effects of genetic and epigenetic aberrations on transcriptional regulators need to be studied further.

Toward this goal, we integrate breast cancer subtype-specific transcriptomic and cistromic (ChIP-seq) datasets to infer regulators and targets underlying breast cancer subtypes. We used 152 breast cancer gene expression datasets adding to a total of 10280 samples, including TCGA (Cancer and Atlas, 2012), METABRIC (Curtis *et al.*, 2012) and patient cohort studies, including one by us (Aure *et al.*, 2013), produced on diverse microarray and RNA-seq platforms, to define subtype-specific genes with a computational search-based algorithm (Zhu *et al.*, 2015). Then relevant regulators of each subtype were identified through the enrichment of two evidence at each subtype’s coexpressed genes: transcription factor (TF) binding evidence from ENCODE ChIP-seq database (Dunham *et al.*, 2012), and motif enrichment evidence within regulatory regions (including TF binding sites and open chromatin regions). Particularly, we found a set of ChIP’d TFs from ENCODE whose binding sites were commonly enriched for loci near coexpressed genes that were upregulated in each subtype, implying that the binding of these TFs is associated in large part with coexpression. Most importantly, we, for the first time, identify a subtype-specific tendency for the mechanism of de-regulation of transcription factors via DNA hypo/hyper methylation in the best prognosis luminal A, or somatic copy number aberrations in the worst prognosis basal subtype. Furthermore, by the integration of ChIP-seq data with expanded coexpressed gene signatures we derive direct evidence of epithelial-mesenchymal transition (EMT), and embryonic stemness in the basal subtype, identifying that target genes are decomposed into those belonging to one, or the other cell lineage, or shared by both lineages (epithelial and stem cell). Finally, based on this analysis we identify two more cell lines, A549 and H1-hESC, as associated to the basal subtype in addition to the known oncogenic MCF10A-Er-Src cell line.

## 2 Materials and methods

To understand how transcriptional events influence the development of breast cancer cells, we reverse engineer the process, starting with coexpressed genes in each luminal A and basal subtype. We used our computational search algorithm SEEK (Zhu *et al.*, 2015) applied to 152 breast cancer datasets (Supplementary Data S1) to accurately identify **extended subtype-specific gene signatures (ESG)** (Supplementary Table S1 and Supplementary Data S2) based on the smaller seed lists of published subtype-specific genes (Muggerud *et al.*, 2006)(Supplementary Table S1). The ESGs were verified to be differentially expressed between subtypes in the external METABRIC cohort (Supplementary Tables S2 and S3).

*ChIP-seq data processing*: We used the collection of ChIP-seq datasets processed by the ENCODE Analysis Working Group. To derive a gene score representing the amount of binding per gene, we first divide the peak signal score (the number of tags) by the 75-percentile peak score of the whole experiment, multiplied by base score 500. Next the peaks’ chromosomal locations are aligned to all gene regions ±50 kb TSS using BEDOPS (Neph *et al.*, 2012) and human hg19. To account for the case when a peak falls within multiple genes’ region, we calculate each gene score as sum of normalized contributions of peak scores: $g=\sum_{f\in P(g)}p(f)/n(f)$ where *g* is the gene score, *f* in *P*(*g*) is the set of peaks in the vicinity of *g*, *p*(*f*) is the peak score of *f*, *n*(*f*) is the number of genes *f* overlaps.

*Cell line prioritization for subtypes*: we used GORILLA (Eden *et al.*, 2009) to compute an enrichment of binding at ESGs in each ChIP-seq experiment that was summarized into gene-based binding scores. For our purpose, we excluded ChIP-seq of CTCF, Rad21, Pol2 and histone marks. This produced 503 experiments for prioritization (Supplementary Data S3 and S4). Then we summarized the results per cell line or cell line group and picked the top cell lines (Supplementary Fig. S1) as models of basal and luminal A.

Next, we identified potential subtype-specific transcription factors based on ChIP-seq data and motif and co-expression evidence, as described below. Detailed steps of TF derivations are in Supplementary Methods.

*ChIP-seq based evidence*: given ESGs in a subtype, we sought ChIP-seq experiments [from ENCODE (Gerstein *et al.*, 2012)] where the sum of binding intensities at the regulatory regions (50 kb ± TSS) of ESGs are significantly higher than binding at random sets of genes. If significance is reached, then ChIP’d TFs are termed regulators of the subtype.

*Motif and coexpression based evidence*: we performed motif analyses within peaks of ChIP-seq experiments in relevant cell lines to search for any motifs that might be enriched, as described previously (Machanick and Bailey, 2011). We additionally required that the TF identified via this analysis must be coexpressed to the target genes, to be considered as a regulator (as a motif often encompasses a family of TFs).

See Supplementary Figure S1 for a workflow of our analyses.

To reveal associations between identified TFs and CNA or DNAme, we obtained relevant datasets from the breast cancer cohort in TCGA (Cancer and Atlas, 2012) and two other independent datasets, METABRIC and OSLO2 (Curtis *et al.*, 2012; Fleischer *et al.*, 2014).

*DNA methylation (DNAme) processing*: TCGA array-based DNAme dataset was downloaded from ICGC in nucleotide-resolution methylation frequencies (beta values). We excluded blood-derived normal samples, and metastatic samples from the TCGA list. We used primary tumor samples and also obtained a separate normal breast tissue sample set from TCGA for comparison. To start, nucleotide-resolution beta values per patient sample was summed to gene-level values. Then, to derive subtype-specific hyper- and hypo-methylated genes, for every gene, we compared the distribution of methylation frequency of subtype patient group and that of the healthy group consisting of normal breast tissue samples, by performing 2-sample *t*-tests with unequal variance with respect to the normal sample group. Genes with *P *<* *0.01 significance are deemed hyper or hypo-methylated respectively.

*Copy number alteration (CNA) processing*: For CNA (Curtis *et al* and TCGA), original data consists of copy number for various chromosomal segments detected per patient. Each copy number ranges from -1 to +1 (in log scale) which is relative to the normal copy number. To derive a gene-based copy number, let *cna* be the copy number of segment *f*; *len* be the number of genes contained in *f*; *F_G_*(*g*) be the set of gained fragments containing gene *g*; *F_L_*(*g*) be the set of lost fragments containing *g*. We calculate:

$$\mathrm{gain}\left(g\right)=\sum_{f\in F_{G}\left(g\right)}\mathrm{cna}\left(f\right)/\mathrm{len}\left(f\right),\;\mathrm{where}\;\mathrm{cna}\left(f\right)>0$$

$$\mathrm{loss}\left(g\right)=\sum_{f\in F_{L}\left(g\right)}\mathrm{cna}\left(f\right)/\mathrm{len}\left(f\right),\;\mathrm{where}\;\mathrm{cna}\left(f\right)<0$$

Then we performed 1-sample *t*-test to obtain genes significantly associated with CNA gains and CNA losses for each tumor subtype.

*Associations between TFs and deregulations*: The rank-based enrichment test GORILLA (Eden *et al.*, 2009) was used to compute the enrichment for ESGs near the top of the rank list of genes sorted by deregulation values. We performed such testing for each CNA and DNAme deregulation. For our context and for each subtype, we combine the gain and loss lists and sort genes by the absolute gain or loss value, giving a rank list of all TFs in genome. Tests that involve TFs used ∼1000 TFs as background. Otherwise, tests involving ESGs used the whole-genome background (∼17 000 genes). *Q*-value procedure (Storey and Tibshirani, 2003) was applied to correct multiple comparisons.

*TF-TF and TF-target networks*: For each ChIP’d TF identified as associated to a subtype, we use its ChIP-seq experiment to identify its binding sites in the ESGs. We connect an edge from ChIP’d TF to a gene in the ESG set if the gene has a higher-than expected peak signal. Iterating this procedure for all ChIP’d TFs results in a TF-target network. TF-TF network results similarly from connecting the ChIP’d TFs to the TF subset of ESGs. For functional enrichment analysis, circled TFs in the network form a seed gene list that was expanded in SEEK, and the top 200 expanded genes were used to determine enriched GO-terms for the TFs.

*Comparisons of basal regions with open chromatin data*: DNase-seq and ATAC-seq were downloaded from ENCODE portal or GEO for MDA-MB231, human mammary epithelial cells (HMEC), H1 embryonic stem cells, mesenchymal stem cells (MSC), A549 and MCF10a-Er-Src cells. Accession IDs were SRR7225842, ENCSR000ELW, ENCSR731QLJ, ENCSR7940FW, ENCSR385SZQ and ENCSR000ENV. ENCODE-provided data were already in narrowPeaks. For MDA-MB231, we processed the data from raw reads by performing alignment (Langmead and Steven, 2013) and peak calling (Zhang *et al.*, 2015) following standard settings. Peak overlap was computed relative to ChIP-seq experiments.

*Decomposition of basal ESGs into lineages*: We assigned basal ESGs to epithelial, mesenchymal and early developmental stem lineages using two strategies. (1) ChIP-seq of A549, MCF10a-Er-Src and H1-hESC. (2) DNase-seq of H1, MSC and HMEC. In (1) experiments of each line form groups that we used to perform a group-based 2-sample *t*-test (unequal variance). Groups were epithelial (A549 and MCF10a-Er-Src) and stem (H1-hESC). This determines whether each gene is significantly assigned to epithelial or stem lineage. In (2) we used log fold-change between cell lines as each cell line is just 1 experiment. Hierarchical clustering was performed, and gene clusters that are specific to each epithelial, stem and mesenchymal were chosen. MSC was used to assign the mesenchymal lineage.

The analysis scripts and data are deposited in Bitbucket https://bitbucket.org/qzhu/breast.cancer.tf/.

## 3 Results and discussion

We inferred regulatory TFs of the subtype-specific extended signature genes (ESGs) from a combination of sources: (i) ChIP-seq experiments and (ii) motif and coexpression evidence. ChIP-seq measures experimental binding while motif, coexpression evidence are designed to expand the scope of inferable TFs from ENCODE by a search of additional motifs that may be overrepresented in the regulatory regions of ESGs (Section 2). Additionally, TFs pointed to by the motifs must be coexpressed with the subtype genes to be reported as regulators in this study. We first performed a global ChIP-seq prioritization as per subtype’s ESGs (Supplementary Data S3 and S4) and identified relevant cell lines of each subtype (Supplementary Fig. S1).

### 3.1 Subtype-specific TFs in luminal and basal subtypes

#### 3.1.1 Transcriptional regulators of the luminal A subtype

**Fig. 1.**
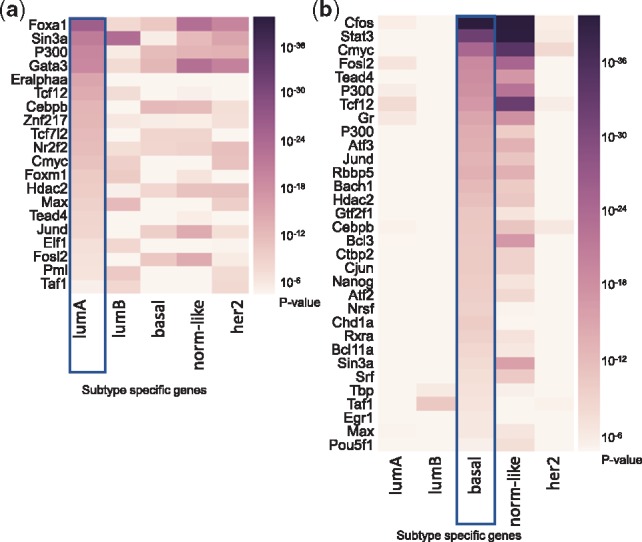
ChIP-seq experiments ranked highest in binding signals at (**a**) luminal A and (**b**) basal extended signature genes (ESGs). Each column indicates one of five ESG sets being interrogated for strength of binding in the ChIP-seq experiments (rows). As expected, lumA-relevant ChIP-seq contains significant binding at lumA genes, but not at basal genes (a)

*ChIP-seq based evidence.* The unbiased search of ChIP-seq experiments, where the sum of binding intensities at the regulatory regions of the subtype-specific ESGs were significantly higher than binding at random genes, identified the three well-known luminal A-associated TFs: Esr1, Gata3 and Foxa1 (Fig. 1). The ChIP-seq experiments identifying these TFs came from the MCF7 and T47D cell lines (Supplementary Fig. S1a), well-accepted models of luminal A biology, supporting our analysis and suggesting that the set of TF targets were subtype-specific (Fig. 1a). In contrast, Gata3, Esr1 and Foxa1 binding was much less significantly enriched in the binding regions of the basal-like ESGs [e.g. difference in –log_10_(*P* value) were 7, 9 and 16 respectively] (Fig. 1a). Other transcription factors with strong target enrichments in luminal A ESGs, but not with basal-like ESGs, were Znf217, Elf1, Myc, Foxm1, Max and Tead4 (Fig. 1a). Altogether, over 20 of the 40 top ChIP-seq experiments (*P *<* *1 × 10^−5^) come from MCF7 (Supplementary Data S3), corroborating with the fact that MCF7 is the most appropriate model for the physiology of luminal A subtype.

*Motif and coexpression based evidence*: Next, by going beyond ChIP-seq data, we found additional regulators of the luminal A ESGs using motif analysis (see Section 2). This returned 16 TFs (Supplementary Tables S4 and S5) that included Xbp1 (X-box binding protein), Pgr (progesterone receptor), Ar (androgen receptor), in addition to the known Foxa1, Esr1, Gata3 for which ChIP-seq data were also available. All of these factors had binding motifs in the 50 kb regulatory regions of luminal A ESGs.

#### 3.1.2 Transcriptional regulators of the basal subtype

When the same analysis was performed for the basal extended signature genes, the ChIP-seq data from the ENCODE revealed significant enrichment for TF binding for Cfos, Stat3, Myc (of MCF10a-Er-Src cells, 20 < –log_10_*P* < 40), Gr, Fosl2, Tcf12, Atf3 (of A549 cells, 15 < –log_10_*P* < 19) and Tead4, Chd1, Jund, Rbbp5, Ctbp2 and several others (of H1-hESC cells, –log_10_*P* > 10) (Fig. 1b), suggesting that these TFs are regulators of the basal subtype. As expected, weak or no enrichment of binding was observed for luminal A ESGs. Additional 23 basal TFs (Supplementary Tables S4 and S5) were identified from expanding the search to include TF motifs and coexpressed TFs in the basal ESGs. These included Bcl11a, Id4, En1, Sox9, for which literature evidence supports their roles in triple-negative breast cancer or as part of the stem cell differentiation program (Adam *et al.*, 2015; Khaled *et al.*, 2015). Among these, the highly relevant basal Foxq1 is a driver of the TGF-beta signaling pathway and participates in crosstalk with Wnt signaling pathway to influence EMT. Overall these results confirm the validity of the inferred TFs.

Intriguingly, two cell lines, A549 and H1-hESC, were identified as associated to the basal subtype in addition to the conventional MCF10a-Er-Src (Supplementary Fig. S1b). This discovery, given the unbiased nature of our analysis, is interesting as A549 is a cell line of basal epithelial origin, while the H1-hESC of stem cell origin. The identification of A549 and H1-hESC as most similar to the basal subtype of breast cancer based on this analysis may be suggestive of a hybrid basal subtype state consistent with the known epithelial and mesenchymal stem cell characteristics of the basal subtype (Sarrió *et al.*, 2008; Wu *et al.*, 2016). Basal specific TFs are found to regulate a large set of basal co-expressed genes, using the TF-TF network described next.

#### 3.1.3 Interaction networks between the TF and their targets in the luminal and basal subtypes

**Fig. 2.**
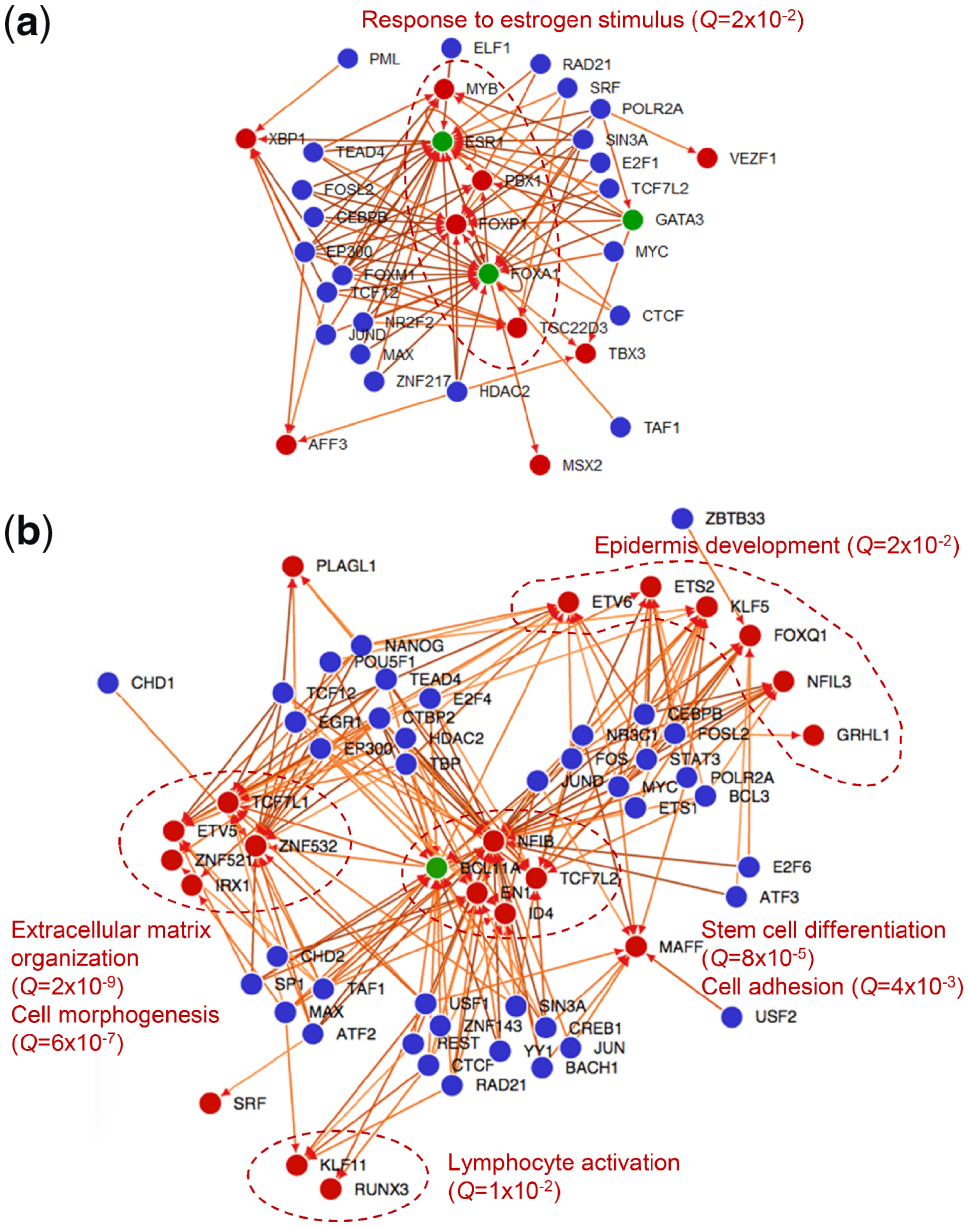
Luminal A (**a**) and basal (**b**) TF-TF network. A directed edge from TF A to TF B means that A binds to B according to ChIP-seq experiment of A in ENCODE database. Blue nodes: ChIP’ed TFs. Red nodes: the TF subset of the subtype’s ESGs which are targeted by the ChIP’ed TFs. TF target genes (red nodes) are partitioned into groups based on different upstream binding factors (blue nodes), and are involved in distinct biological processes (circled and process names in red). In addition to this network, we show an extended TF-target network as a heatmap in Supplementary Figures S3 and S4

In the next step, we connected the above inferred TFs to the subtype’s ESGs based on the evidence for binding. In doing so, we created a directed TF-TF binding network (Fig. 2) through which we analyzed the interactions between these identified TFs and their targets in the subtype-specific extended signature genes (Fig. 2). A directed edge from TF A to TF B in this network means that A binds to B according to ChIP-seq experiment of A in ENCODE database. The ChIP’ed TFs are highlighted in blue, while the TF subset of the subtype’s co-expressed genes which are targeted by the ChIP’ed TFs are highlighted in red. TF target genes (red nodes) are partitioned into groups based on different upstream binding factors, i.e. the ChIP’ed TFs (blue nodes), according to the distinct biological processes they are involved in. In addition to this network, we show an extended TF-target network as a heatmap in Supplementary Figure S3, including non-TF targets. In the case of luminal A TF network (Fig. 2a), the self- and co-regulatory functions of ESR1, FOXA1 and GATA3 have been well described (Hurtado *et al.*, 2011). Further examining the targets of this network (Supplementary Fig. S3), we found binding sites were enriched near genes including FOXA1, ESR1, as well as EVL, PREX1, KCTD3, VAV3 and many others (Supplementary Fig. S3). Among these, KRT18, SIAH2, TFF1 contain ER-alpha binding sites in distal regulatory regions with sites overlap with sites of chromatin interactions reported in MCF7 ChIA-PET (Fullwood *et al.*, 2009), confirming their regulatory roles.

Luminal A suggests a simple network that is dominated by estrogen response, where the majority of regulators target ESR1 and FOXA1. In the basal subtype, however, downstream genes (red nodes) can be seen distinctly partitioned into groups according to unique partners of upstream factors (blue nodes) (Fig. 2b). These downstream groups are highly functionally distinct (see biological process annotations in Supplementary Table S6), and further suggestive of the heterogeneous, dual EMT nature of the basal subtype. Indeed, the regulatory function of basal TFs partitions direct binding targets into two groups (Supplementary Fig. S4): those found in 1) epithelial lineage: ERRFI1, ANXA1, EDN1, MID1; and 2) stem cell lineage: BCL11A, LPHN2, ROR1, ZNF532, GCNT2, PODXL, SPRY2, EPHB3. Multiple biological processes in the complex TF-TF network (Fig. 2b) confirm the hallmark of EMT in the basal subtype (Wu *et al.*, 2016).

To investigate whether sites indicated by ChIP-seq peaks of A549 and H1-hESC TFs are active in the basal subtype, we gather data for open-chromatin regions in a true basal cell line MDA-MB-231. We compared the peaks of Tead4, Max, Hdac2 ChIP-seq in H1-hESC (3 of the most enriched experiments for basal ESGs) with the ATAC-seq of MDA-MB-231. Pooled peaks of these TFs represent 45% of all open-chromatin peaks in MDA-MB-231 (Supplementary Table S7). For A549, ChIP-seq peaks of Cebpb, Fosl2, Tcf12 together recover 40% of peaks in ATAC-seq; for MCF10a-Er-Src it is over 55% (Supplementary Table S7). Overall, these results suggest that there are shared regulatory programs and regions between A549, embryonic cell lines and the basal subtype.

#### 3.1.4 Extended analysis of EMT and stemness in the basal breast cancer

The basal ESGs contain vimentin (VIM, coexpression *P *=* *0.0351), c-Met oncogene (MET, *P *=* *0.0071), FOXQ1 (*P *=* *0.0008) which are EMT hallmarks and regulators. We examined a public Zeb1 ChIP-seq sample (GSM2360622) to ask how basal ESGs are targeted by Zeb1, which represses E-cadherin and is a key inducer of EMT. Indeed, basal ESGs are significantly enriched in Zeb1 targets (*P *=* *7.67 × 10^−29^). We hypothesize that the basal subtype contains mixed cell states and populations marked by the distinct expression of lineage-specific genes for multiple lineages. To this end, we decompose basal ESGs (also a mixture of genes) into those belonging to one, or the other cell lineage, or both lineages (epithelial and stem cell) (Supplementary Figs S4 and S5). We focus on a subset of 265 ESGs that have substantial genome-wide TF binding. We used two approaches. First, TF ChIP-seq experiments may divide the basal ESGs based on differential TF binding at these genes in epithelial (A549, MCF10a-Er-Src) and stem cells (H1-hESC). We note that 79, and 95 genes have TF binding uniquely associated with epithelial (*P *<* *0.05), and embryonic stem lineage (*P *<* *0.05) respectively (Supplementary Fig. S4). 91 genes cannot be distinguished between the two (Supplementary Fig. S4). Second, the same ESGs were decomposed based on open chromatin peaks in DNase I hypersensitivity sequencing data in HMEC, MSC and H1-hESC cells (Supplementary Fig. S5a). Overlap of the two approaches show 44 embryonic stem, 25 epithelial and 15 mesenchymal genes within the basal ESGs (Supplementary Fig. S5b). These groups participate in specific biological functions according to the GO-term analysis (Supplementary Fig. S5c).

#### 3.1.5 siRNA, knockout experiments of BCL11A, CTBP2, REST, FOXA1 support the regulation of ESGs

To experimentally verify that these identified TFs indeed regulate ESGs, we obtained public transcriptomic datasets from TF knockout and siRNA conditions for 4 TFs from basal and luminal A regulator lists: BCL11A, CTBP2, REST, and, FOXA1. For each of the luminal A and basal subtypes we found that a greater proportion of subtype-specific genes and subtype-specific TFs are perturbed (i.e. fold-change > 1.5) upon TF knockdown or knockout, when compared to a random set of genes of matched size (Supplementary Fig. S6). Overall this suggests that the regulators identified computationally exert an effect on the subtype-specific target genes also in experimental setting.

### 3.2 Tendencies of TFs to reside in regions affected by CNA and DNAme suggest distinct mechanisms of dysregulations for subtypes

Development of cancer involves dysregulation at multiple levels, including changes to the DNA, such as somatic CNA and aberrant promoter DNAme. We therefore assessed whether in the different breast cancer subtypes the sets of cancer subtype-specific TFs described here, were more often subjected to genetic (CNA) and/or epigenetic (DNAme) aberrations than random sets of TFs. To address this, we used data from 700 breast tumors with subtype-specific DNAme, and CNA data from TCGA and METABRIC. We summarized aberrations on a gene-level to facilitate comparisons with the ESGs, and tested the relevance of each type of subtype-specific aberration (CNA, DNAme) on the subtype-specific regulators.

#### 3.2.1 Groups of TFs and targets are distinctly associated with subtype-specific breast cancer aberrations including DNAme, CNA

Our results (Table 1) indicate that the groups of subtype-specific TFs identified by our method are more likely perturbed by different cancer aberrations than expected for random TFs. In luminal A tumors, luminal A TFs and their targeted genes are significantly dysregulated by DNAme (see TCGA and Fleischer *et al.* in Table 1) and to a much less extent by CNA (see TCGA, Curtis *et al.* in Table 1) (note that Curtis *et al.* and Fleischer *et al.* are external cohorts provided in addition to TCGA). In the basal subtype, basal TFs and their targeted genes are dysregulated by CNA (see TCGA, Curtis *et al.* in Table 2), but not by DNAme (see TCGA, Fleischer *et al.* in Table 1). Patterns of CNA (Supplementary Fig. S7b) support this observation. The results reflect distinct mechanisms of dysregulation depending on the subtype examined with a distinct pattern of CNA targeting of basal TFs in the basal subpopulation.

**Table 1.** Significance of accumulation of CNA, DNAme at luminal and basal TFs^b^

Dataset	Type	Basal TFs in basal tumors (*Q*-val)	Basal targets^a^ in basal tumors (*Q*-val)	LumA TFs in lumA tumors (*Q*-val)	LumA targets^a^ in lumA tumors (*Q*-val)
TCGA	CNA	0.00214	0.00195	0.04	0.023
Curtis *et al.*	CNA	0.00534	0.00018	0.015	0.085
TCGA	DNAme	0.569	0.296	0.022	0.00013
Fleischer *et al.*	DNAme	0.3109	0.0939	0.0153	0.00011

a Genes that overlap between ChIP-seq targets, extended signature genes.

b Shaded regions indicate consistent patterns of dysregulation across cohorts.

Patterns of DNAme dysregulation at the individual TF level are particularly subtype-specific (Supplementary Fig. S7a). For example, we located a subset of TFs containing stem cell differentiation factors SOX9, EN1, GRHL1, FOXC1, ETS2, ETV6 which are hypomethylated in basal tumors and hypermethylated in luminal A tumors (Supplementary Fig. S7b). Such factors in hyper/hypomethylation states possibly suggest that stem-like properties are effectively suppressed in the non-basal subtypes through DNAme. On the other hand, luminal A TFs are characterized by hypomethylation in luminal A/B tumors (Supplementary Fig. S7a). Evidence of hypomethylation marks is noted at GATA3, BHLHE40, ZBTB42, SPDEF, TOX3 in luminal A patients and not in basal (Supplementary Fig. S7a). Importantly, CpGs located within Foxa1 ChIP-seq peaks near luminal A targets have clear lower methylation than normal and basal groups (Supplementary Fig. S8), suggesting distinct methylation may facilitate the usage of TF binding region in a subtype specific manner. Thus, differential DNAme at TFs plays a critical role in maintaining luminal progenitor states in luminal cancers and epithelial and stem cell states in basal cancers.

## 4 Conclusion

This study demonstrates that each subtype is regulated by a unique repertoire of TFs, and that these subtypes differ by the unique combinations of TFs, switching of binding sites and binding partners. Therefore, forces that deregulate this transcriptional network, through mechanisms of CNA or DNAme of the TFs and their targets, are plausible causes of how subtypes arise. Understanding the interplay between DNAme and CNA will lead to a better understanding of how subtypes arise. The groups of TFs and their targets described here revealed an unexpected tendency to copy number aberrations and DNAme, suggesting that distinctive mechanisms underlie transcriptional regulation. Importantly, luminal A TFs tended toward DNA hypomethylation in luminal cancers while basal TFs tended toward CNA gains/losses in basal cancers. Consistent with the latter finding, basal breast cancer cells accumulate a large number of CNAs, which cause genomic instability (Weigman *et al.*, 2012), and exhibit highly dynamic and complex phenotypes. In luminal A cancer, the hypomethylation enhances the commitment of luminal lineage at key lineage-specific markers (GATA3, ESR1, FOXA1). Moreover, hypomethylation largely extends to the TF binding regions and enhancers of these lineage markers (Fleischer *et al.*, 2017), suggesting a mechanism by which DNAme may control gene expression by interfering with transcription factor binding.

In contrast to luminal A, the TFs driving the basal subtype have remained largely unknown. For this reason there are no ChIP-seq data for ER negative breast cancer cell lines either. Here we report A549, H1-hESC, MCF10a-Er-Src (prioritized from cell lines of 503 ENCODE ChIP-seq) could be used to derive a list of TFs connected in gene regulatory networks in the basal subtype, delineating TFs and their targets and show that they are related to the EMT process and stemness property. The specific TFs and regulatory targets in the basal subtype are first reported here. We have been able to divide these genes as belonging to one of distinct lineages (epithelial, mesenchymal and stem cell) through our network, and summarized TF binding and open chromatin regions.

Applying the combined ChIP-seq/SEEK method also enabled to decompose the heterogeneity among the basal extended signature genes. These genes, otherwise indistinguishable due to their coexpression, have been thanks to the TF binding analysis decomposed into genes belonging to epithelial and to stem cell lineages by the use of A549 and H1-hESC ChIP-seq experiments. A549 and H1-hESC cell lines resemble cell populations in the basal subtype. Of note, H1-hESC is an embryonic cell line. The embryonic stem cell population may be related to the fetal mammary stem cells reported prior (Wahl and Spike, 2017), which are also effectively ER^–^, PR^–^ and Her2 low, same as in basal-like breast cancer. Overall, the similarity to these two cell lines shows that there are at least two subpopulations in the basal tumors which are associated with embryonic stemness, epithelial and mesenchymal transitional process. Thus, using ENCODE ChIP-seq samples from a diverse panel of cell lines enabled us to postulate unique tumor cell types located within the heterogeneous basal subtype.

## Supplementary Material

btz709_Supplementary_Data
