# Supplementary material for: Subtype-specific transcriptional regulators in breast tumors subjected to genetic and epigenetic alterations
Source: Bioinformatics. 2019 Sep 16;36(4):994–9. doi: 10.1093/bioinformatics/btz709 (PMC7031777; doi:10.1093/bioinformatics/btz709)
Supplement: btz709_Supplementary_Data [file btz709_supplementary_data.zip › All.Supplementary.Figures.and.Tables.pdf]

Supplementary Materials (Supplementary Figures & Tables)

Supplementary Figures

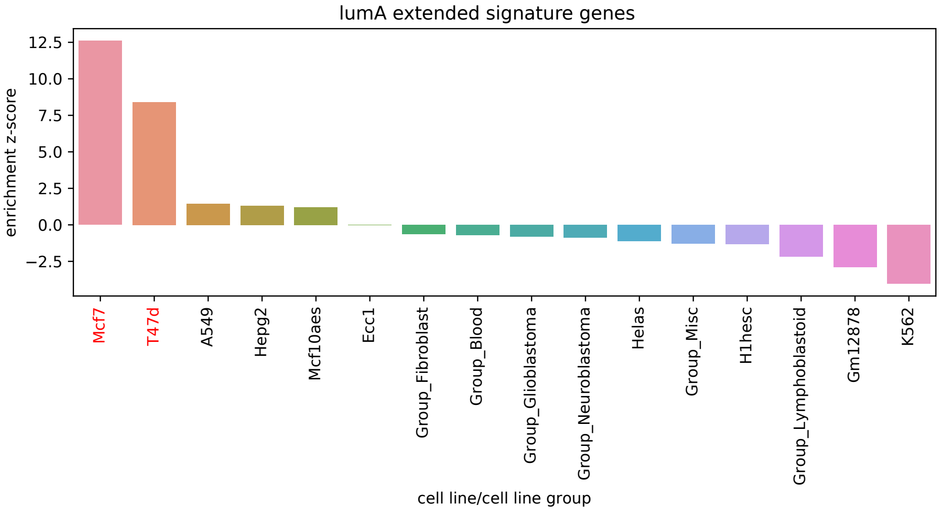

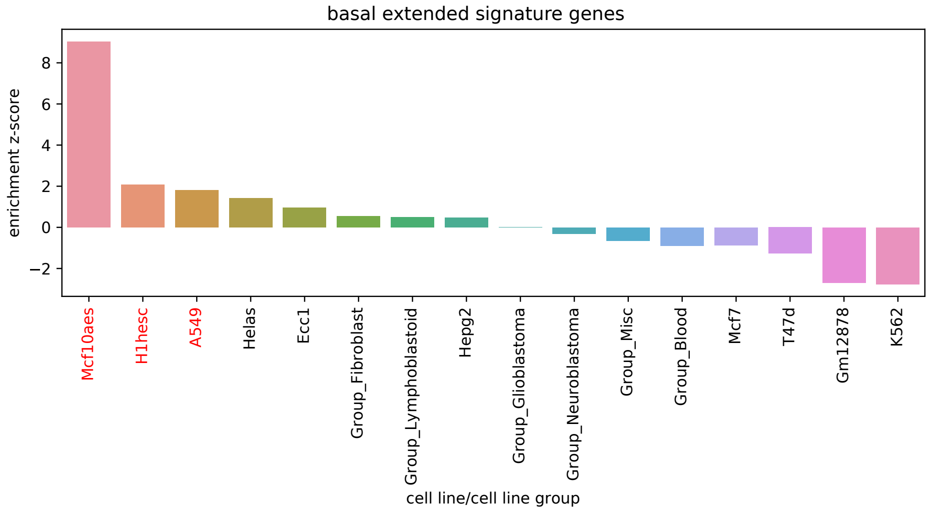

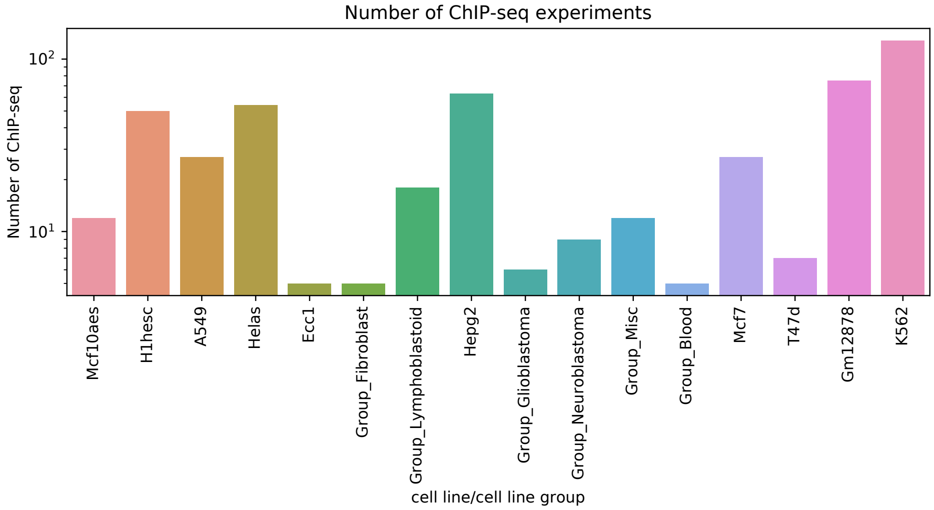

Supplementary Figure 1: Prioritization of TF ChIP-seq experiments in ENCODE based on subtype ESGs, excluding Ctcf, Rad21, Pol2, and histone mark experiments. Individual ChIP-seq's enrichment of peaks near subtype-specific ESGs was computed (Supplementary Table 1), and then the enrichment results were summarized by cell line or by cell line group. Cell lines that have less than 5 ChIP-seq experiments each were grouped together. A PAGE enrichment score was next computed. (a) Luminal A ESGs, (b) Basal ESGs. (c) Number of ChIP-seqs per cell line. Red font indicates relevant cell lines of each subtype. (Groups in the chart. Group_Lymophoblastoid: Gm08714, Gm10847, Gm12891, Gm12892, Gm15510, Gm18505, Gm18526, Gm18951, Gm19099, Gm19193; Group_Blood: Dnd41, Nb4, Pbde, Pdefetal, Raji; Group_Glioblastoma: Gliobla, U87, Pfsk1, Nha; Group_Misc: U2os, Panc1, Hmec, Hsmmt, Nt2d1, Hct116, Hek293, Huvec; Group_Fibroblast: Progfib, Imr90, Nhdfad, Nhlf, Nhek; Group_Neurblastoma: Shsy5y, Sknmc, Sknsh, Sknshra.)

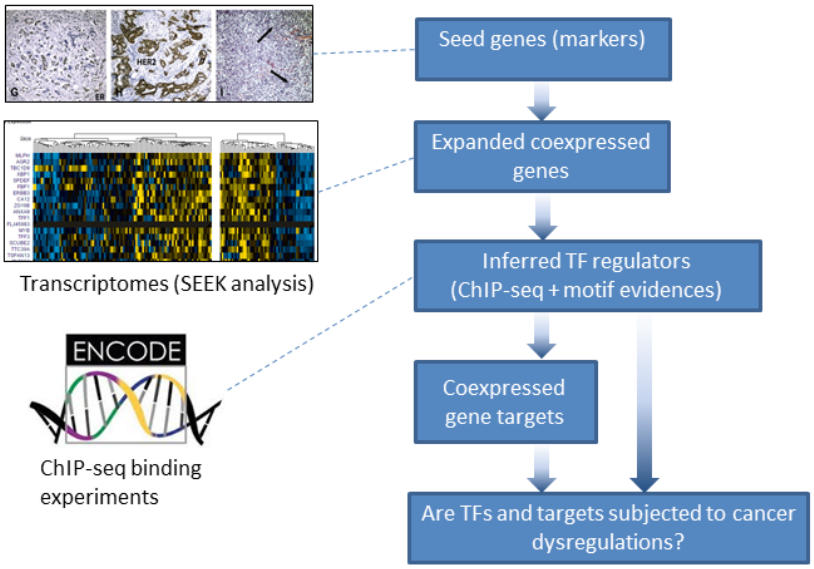

Supplementary Figure 2: Schematic of the workflow. As a first step, SEEK coexpression analysis enlarges the subtype specific seed genes to a larger signature gene set (termed extended signature genes). Then, TFs that may regulate these genes were identified. There are two sources of data used in our analysis, namely ENCODE ChIP-seq experiments (marked with #1) and motif-based analysis of cis-regulatory ChIP-seq sequences of extended signature genes (marked with #2). They together help reveal distinct regulators of subtype signature genes. Afterward, to validate the TF regulators, we ask whether they are more often than random subjected to breast cancer subtype specific dysregulations (copy number aberrations and DNA methylations).

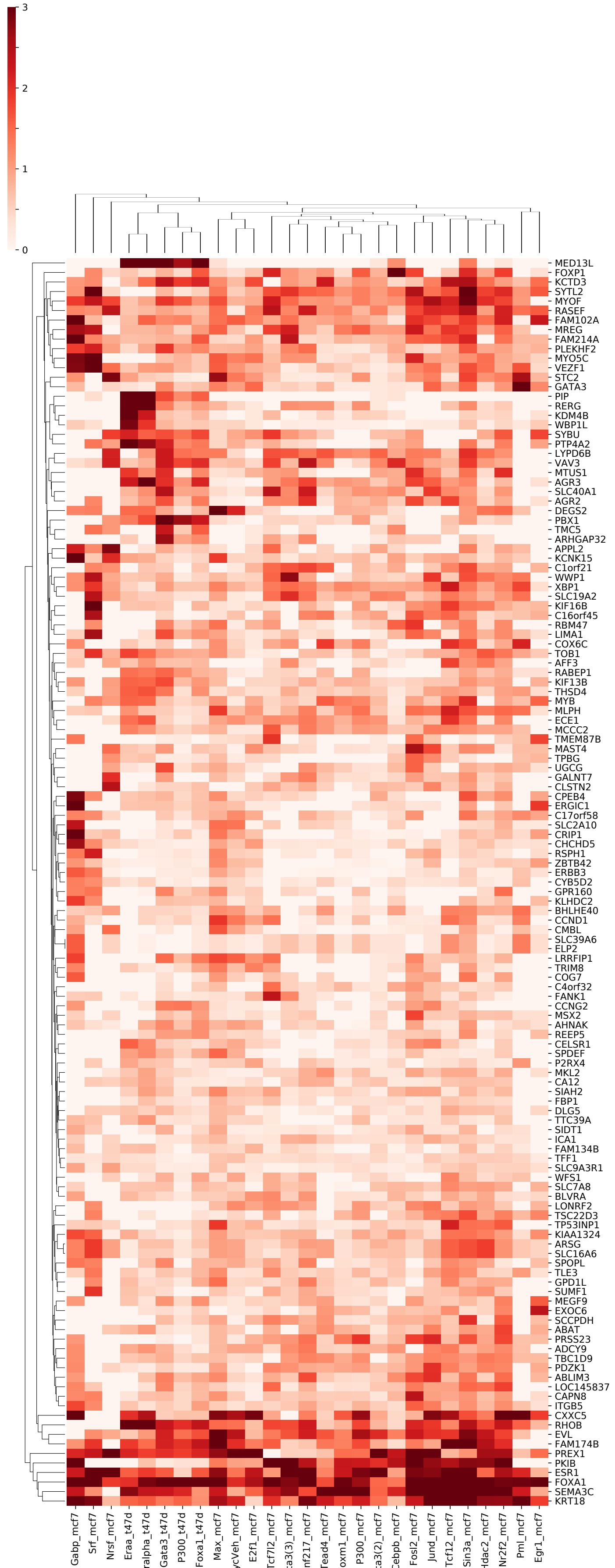

0
1
2
3
MED13L
FOXP1
KCTD3
SYTL2
MYOF
RASEF
FAM102A
MREG
FAM214A
PLEKHF2
MYO5C
VEZF1
STC2
GATA3
PIP
RERG
KDM4B
WBP1L
SYBU
PTP4A2
LYPD6B
VAV3
MTUS1
AGR3
SLC40A1
AGR2
DEGS2
PBX1
TMC5
ARHGAP32
APPL2
KCNK15
C1orf21
WWP1
XBP1
SLC19A2
KIF16B
C16orf45
RBM47
LIMA1
COX6C
TOB1
AFF3
RABEP1
KIF13B
THSD4
MYB
MLPH
ECE1
MCCC2
TMEM87B
MAST4
TPBG
UGCG
GALNT7
CLSTN2
CPEB4
ERGIC1
C17orf58
SLC2A10
CRIP1
CHCHD5
RSPH1
ZBTB42
ERBB3
CYB5D2
GPR160
KLHDC2
BHLHE40
CCND1
CMBL
SLC39A6
ELP2
LRRFIP1
TRIM8
COG7
C4orf32
FANK1
CCNG2
MSX2
AHNAK
REEP5
CELSR1
SPDEF
P2RX4
MKL2
CA12
SIAH2
FBP1
DLG5
TTC39A
SIDT1
ICA1
FAM134B
TFF1
SLC9A3R1
WFS1
SLC7A8
BLVRA
LONRF2
TSC22D3
TP53INP1
KIAA1324
ARSG
SLC16A6
SPOPL
TLE3
GPD1L
SUMF1
MEGF9
EXOC6
SCCPDH
ABAT
PRSS23
ADCY9
TBC1D9
PDZK1
ABLIM3
LOC145837
CAPN8
ITGB5
CXXC5
RHOB
EVL
FAM174B
PREX1
PKIB
ESR1
FOXA1
SEMA3C
KRT18
Gabp_mcf7
Srf_mcf7
Nrsf_mcf7
Eraa_t47d
Eralpha_t47d
Gata3_t47d
P300_t47d
Foxa1_t47d
Max_mcf7
CmycVeh_mcf7
E2f1_mcf7
Tcf7l2_mcf7
Gata3(3)_mcf7
Znf217_mcf7
Tead4_mcf7
Foxm1_mcf7
P300_mcf7
Gata3(2)_mcf7
Cebpb_mcf7
Fosl2_mcf7
Jund_mcf7
Tcf12_mcf7
Sin3a_mcf7
Hdac2_mcf7
Nr2f2_mcf7
Pml_mcf7
Egr1_mcf7

Supplementary Figure 3: Luminal A targets of TF regulators. Rows: top luminal A extended signature genes that are targets of luminal A-specific TF ChIP-seq experiments. Columns: TF regulators, ChIP'd from ENCODE with cell lines indicated. Entry in heatmap: binding signals of a TF at the upstream region 50kb of an extended signature gene.

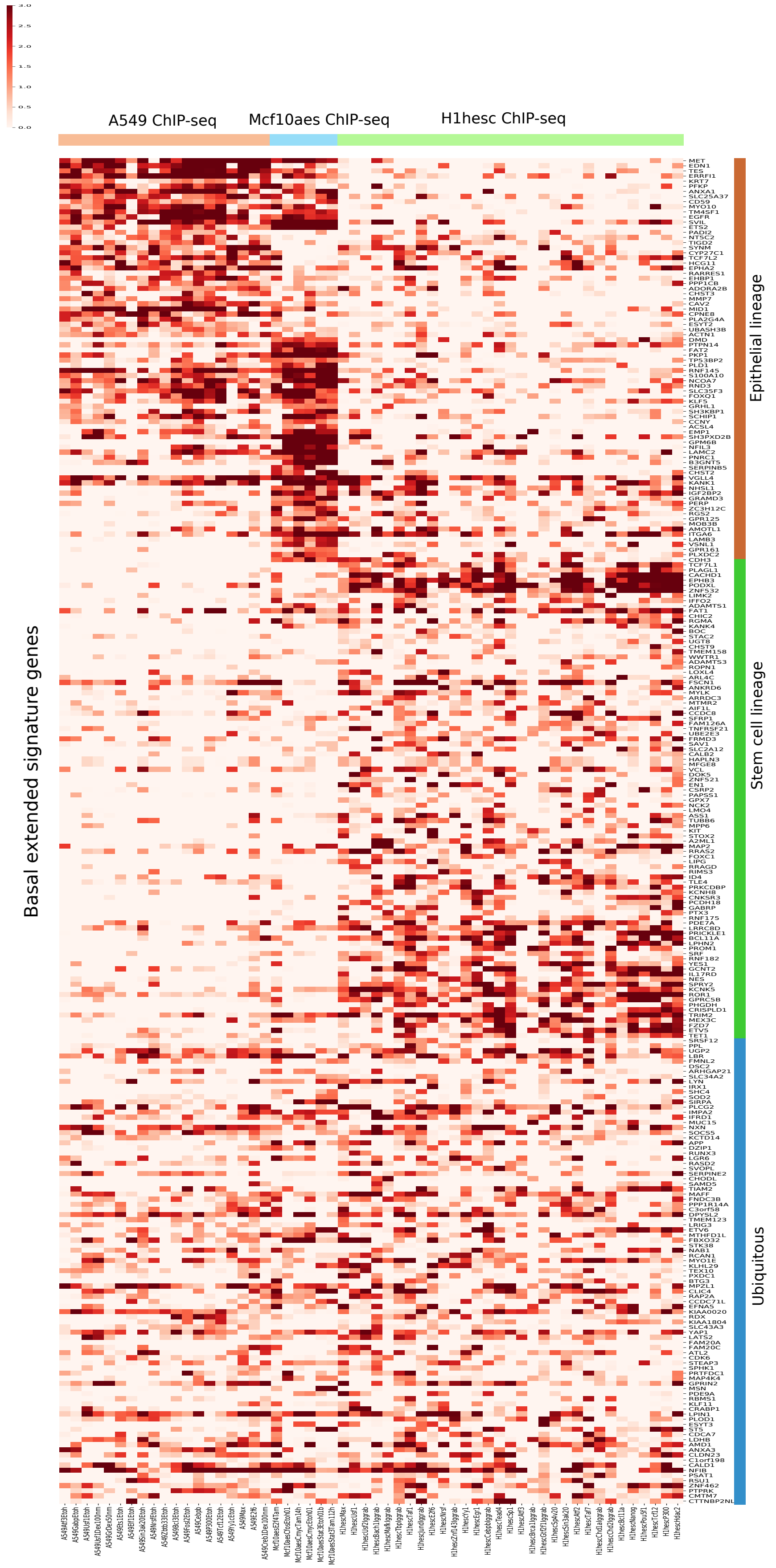

3.0
2.5
2.0
1.5
1.0
0.5
0.0
A549 ChIP-seq
Mcf10aes ChIP-seq
H1hesc ChIP-seq
Basal extended signature genes
Epithelial lineage
Stem cell lineage
Ubiquitous

Supplementary Figure 4: Decomposition of basal ESGs into epithelial and stem cell lineages using A549, MCF10A-er-src, and H1hesc **ChIP-seq experiments**. ChIP-seq experiments from these cell lines form groups with which we compute 2-sample t-test between groups for each ESG gene. Shown are significant genes. Epithelial lineage genes: significant in A549+MCF10A-er-src experiment group ($P<0.05$). Stem cell lineage genes: significant in H1hesc experiment group ($P<0.05$). Ubiquitous: present in both lineages but not significant in any of comparisons.

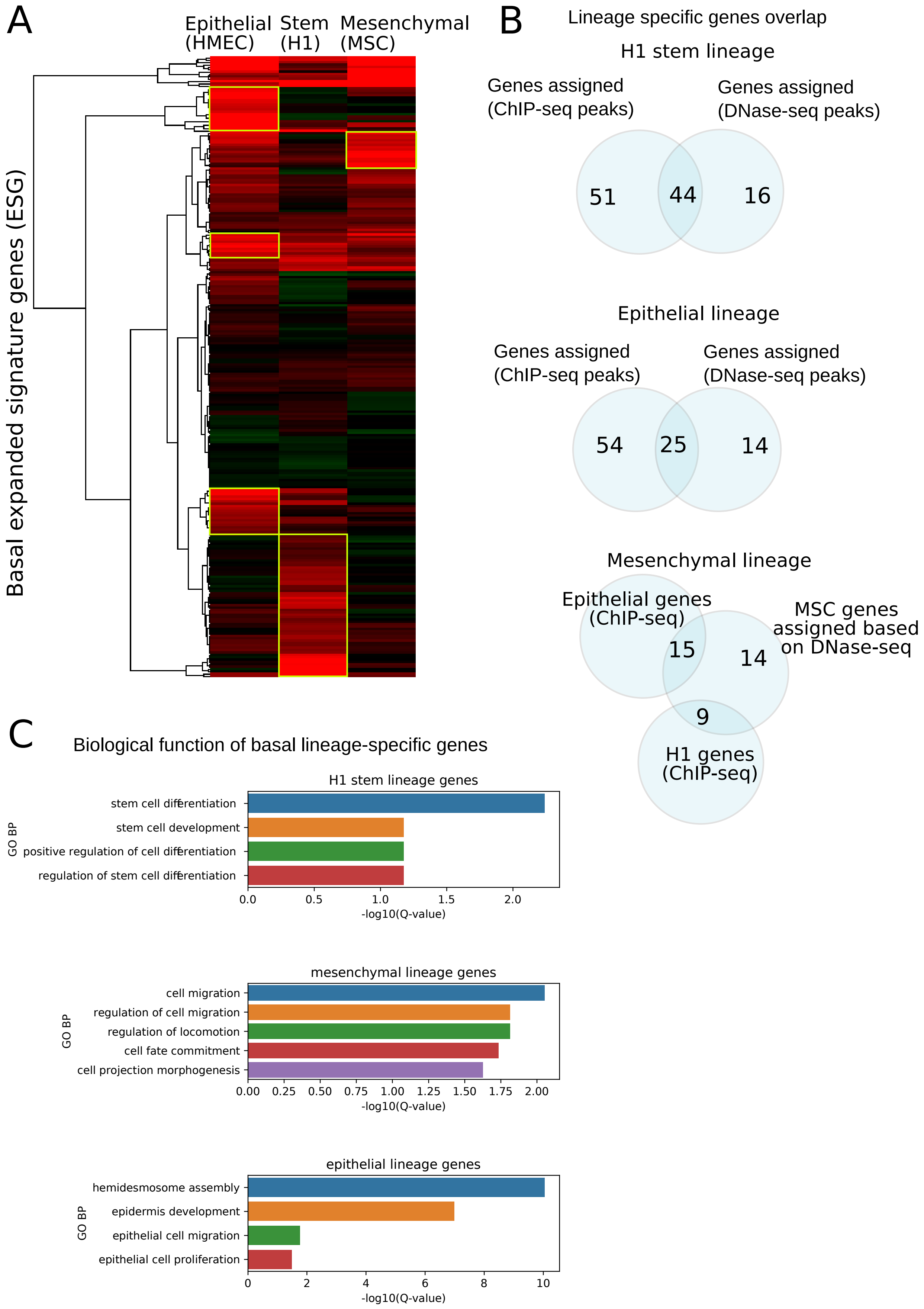
A
Epithelial (HMEC)
Stem (H1)
Mesenchymal (MSC)
Basal expanded signature genes (ESG)
B
Lineage specific genes overlap
H1 stem lineage
Genes assigned (ChIP-seq peaks)
Genes assigned (DNase-seq peaks)
51
44
16
Epithelial lineage
Genes assigned (ChIP-seq peaks)
Genes assigned (DNase-seq peaks)
54
25
14
Mesenchymal lineage
Epithelial genes (ChIP-seq)
MSC genes assigned based on DNase-seq
15
14
9
H1 genes (ChIP-seq)
C
Biological function of basal lineage-specific genes
H1 stem lineage genes
GO BP
stem cell differentiation
stem cell development
positive regulation of cell differentiation
regulation of stem cell differentiation
0.0
0.5
1.0
1.5
2.0
-log10(Q-value)
mesenchymal lineage genes
GO BP
cell migration
regulation of cell migration
regulation of locomotion
cell fate commitment
cell projection morphogenesis
0.00
0.25
0.50
0.75
1.00
1.25
1.50
1.75
2.00
-log10(Q-value)
epithelial lineage genes
GO BP
hemidesmosome assembly
epidermis development
epithelial cell migration
epithelial cell proliferation
0
2
4
6
8
10
-log10(Q-value)

Supplementary Figure 5: Decomposition of basal ESGs into epithelial, stem cell, and mesenchymal lineages using **open chromatin DNase-seq** of mammary epithelial cells (HMEC), mesenchymal stem cells (MSC), and embryonic stem cells (H1). (a) Hierarchical clustering of basal ESGs in the 3 open chromatin datasets, using same basal ESG genes as Supplementary Figure 4. (b) Overlap between lineage-specific genes assigned by using ChIP-seq (Supplementary Figure 4) and DNase-seq data. (c) GO biological process enrichment of the overlapping lineage-specific genes in (b): H1 stem (N=44 genes), epithelial (N=25), mesenchymal (N=14). Enrichment was obtained by expanding each gene-set in SEEK and next performing a GO-term analysis on the expanded gene-set.

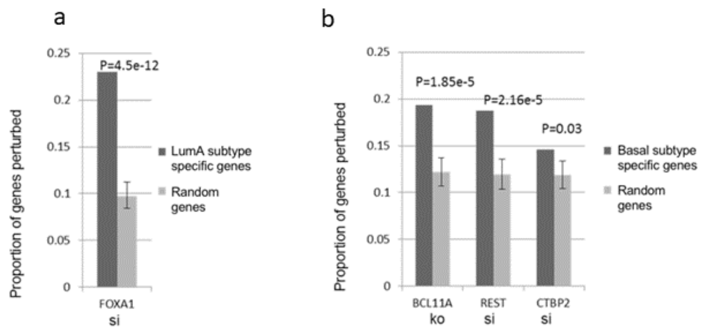

Supplementary Figure 6: Effect of TF knockout or siRNA knockdown on subtype specific genes, subtype specific TFs, and randomly selected genes. We count the number of genes perturbed in each case. A gene is perturbed if abs(FCH)>$\mu+\sigma$ where FCH is fold-change relative to a control condition without perturbation, μ is the mean and σ is the standard deviation of expression fold-change. (a–b) Basal regulators: BCL11A (knock out), REST (siRNA), CTBP2 (siRNA).(c–d) Luminal A regulator FOXA1 perturbation (siRNA).

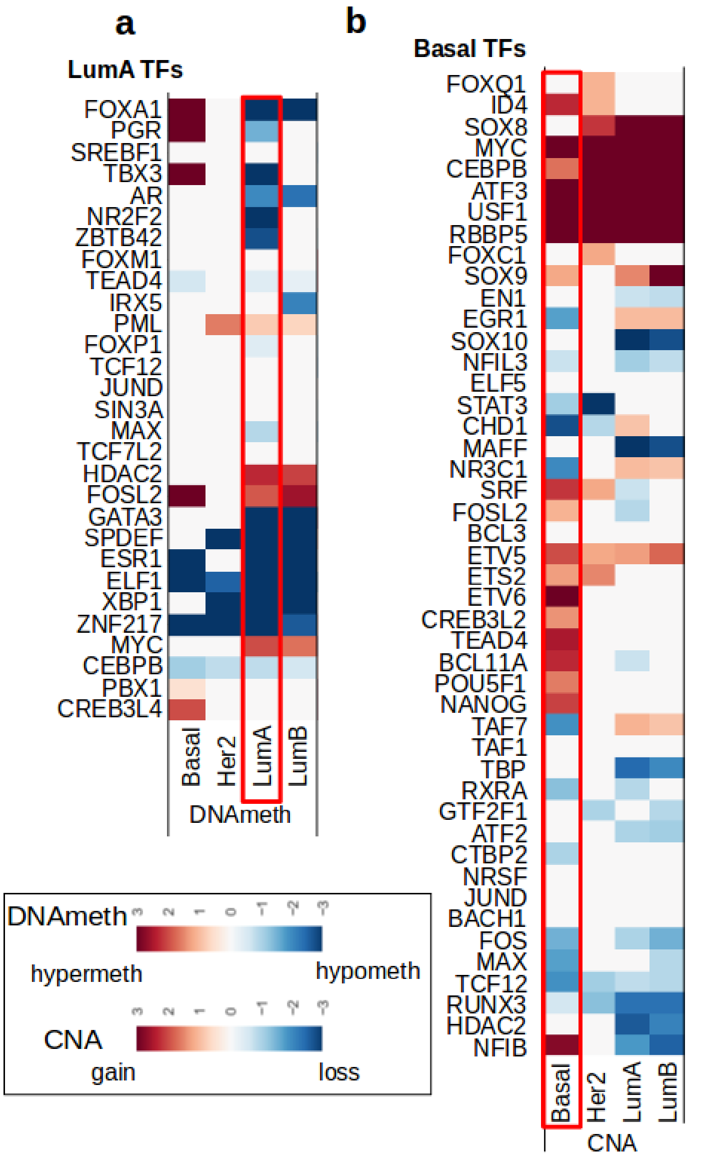

Supplementary Figure 7: Deregulation patterns based on TCGA data. (a) Luminal A TFs exhibit tendencies to DNA methylations. (b) Basal TFs exhibit tendencies to copy number aberrations (CNA). Within each (a) and (b), each column in the heatmap represents a tumor subtype where the deregulation is scored for the respective TF genes. The boxed red highlights the tumor subtype to focus in each panel.

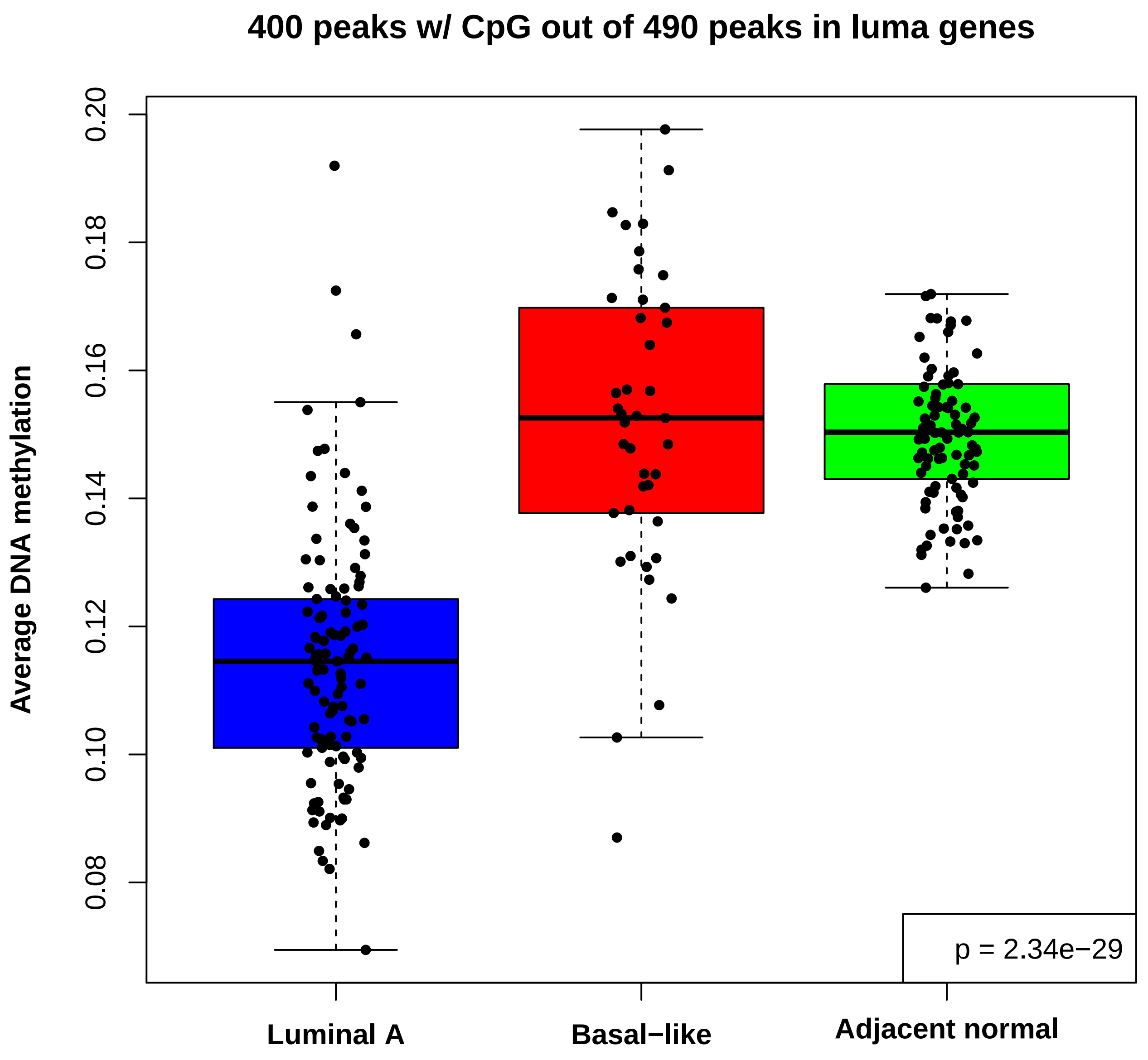

Supplementary Figure 8: FOXA1 ChIP-seq peaks' DNA methylation status to Luminal A ESG genes.

Supplementary Tables

(a)

**Subtype and source of genes**	**Seed Genes**
Luminal A (Muggerud *et al.*, 2006)	ENPP5 TCEA3 NPNT SNX13 STEAP2 MGRN1 CYP2A6 KIAA0182 ECHDC2 FMO5 SELENBP1 MUC1 CRAT CFB RARRES3 CYB5A GALNT10 HSD17B4 APPL2 PTP4A2 ASAH1 ALCAM MSX2 SLC40A1 SVEP1 SNED1 PLAT FMOD ADRA2A ECE1 BCAM SHC2 ACBD4 GSTM3 CAMK2N1 RALGPS1 PTPRN2 BLVRA AGTR1 NPY1R TLE3 PHF15 MED13L CCND1 QDPR SIAH2 COX6C SCNN1A TFF3 MCCC2 FBP1 ANXA9 REEP5 LRBA HEXIM1 BECN1 TCEAL1 RERG SLC39A6 RABEP1 ESR1 ACADSB VAV3 NAT1 SCUBE2 GATA3 FOXA1 XBP1
Luminal B (Parker *et al.*, 2009)	CDC6 CCNB1 UBE2T NUF2 BLVRA SLC39A6 ESR1 CXXC5
Her2 (Muggerud *et al.*, 2006)	TBPL1 TLK1 FLOT2 SMARCE1 MED24 STARD3 GRB7 ERBB2 S100P CEACAM6
Basal-like (Muggerud *et al.*, 2006)	ZNF532 B3GNT5 CDK6 KDSR NCL SLC5A6 CHI3L2 SLPI CXCL1 VGLL1 DSC2 FOXC1 MFGE8 ACTG2 GABRP TRIM29 KRT5 KRT17 CX3CL1 CDH3 SGCE FZD7 VCL EXT2
Normal-like (Muggerud *et al.*, 2006)	KRT13 RAPGEF3 RAB11FIP5 ACSS2 GNB2L1 TFAP2C GSTA4 CA2 AQP3 AKR1C1 ACSL1 LTF PIK3R1 ABLIM1 PTPRM PAM

(b)

**Subtype**	**Links to SEEK analysis (containing extended signature genes)**
Luminal A SEEK analysis	http://seek.princeton.edu/viewer33.jsp?sessionID=1520269952792&sort_sample_by_expr=true<br>Coexpressed genes (includes P-values for all genes):<br>**http://seek.princeton.edu/servlet/GetScoreServlet?sessionID=1520269952792&type=gene_score&keyword=all_sorted&show_query=true**
Luminal B SEEK analysis	http://seek.princeton.edu/viewer33.jsp?sessionID=1520270226536&sort_sample_by_expr=true<br>Coexpressed genes (includes P-values for all genes):<br>**http://seek.princeton.edu/servlet/GetScoreServlet?sessionID=1520270226536&type=gene_score&keyword=all_sorted&show_query=true**
Basal-like SEEK analysis	http://seek.princeton.edu/viewer33.jsp?sessionID=1520270648156&sort_sample_by_expr=true<br>Coexpressed genes (includes P-values for all genes):<br>**http://seek.princeton.edu/servlet/GetScoreServlet?sessionID=1520270648156&type=gene_score&keyword=all_sorted&show_query=true**
Normal-like SEEK analysis	http://seek.princeton.edu/viewer33.jsp?sessionID=1520270901440&sort_sample_by_expr=true<br>Coexpressed genes (includes P-values for all genes):<br>**http://seek.princeton.edu/servlet/GetScoreServlet?sessionID=1520270901440&type=gene_score&keyword=all_sorted&show_query=true**
Her2-enriched SEEK analysis	http://seek.princeton.edu/viewer33.jsp?sessionID=1520270391563&sort_sample_by_expr=true<br>Coexpressed genes (includes P-values for all genes):<br>**http://seek.princeton.edu/servlet/GetScoreServlet?sessionID=1520270391563&type=gene_score&keyword=all_sorted&show_query=true**

Supplementary Table 1: Seed genes per subtype (a), and the corresponding SEEK coexpression expansion (b).

(a)

	Total expanded genes	Number of expanded genes found to be differentially expressed in METABRIC per P-value cutoff			
		P<0.05	P<0.01	P<0.001	P<1e-5
Her2	411	324	300	277	235
LumA	459	445	442	439	427
LumB	486	475	471	470	456
Normal	456	398	375	348	299
Basal	471	464	461	458	450
Her2-top 100	*94*	*81*	*77*	*74*	*65*

(b)

	Total expanded genes	Number as a percentage of total expanded genes			
		P<0.05	P<0.01	P<0.001	P<1e-5
Her2	411	0.788	0.730	0.674	0.572
LumA	459	0.969	0.963	0.956	0.930
LumB	486	0.977	0.969	0.967	0.938
Normal	456	0.873	0.822	0.763	0.656
Basal	471	0.985	0.979	0.972	0.955
Her2-top 100	*94*	*0.862*	*0.819*	*0.787*	*0.691*

Supplementary Table 2: Fraction of extended signature genes (ESGs) that are differentially expressed in the METABRIC cohort subtypes. For each ESG gene, we computed the differential expression t-test statistics of the gene across intrinsic subtype groups in METABRIC. Number of ESGs that are significant, as per 0.05, 0.01, 0.001, and 1e-5 P-value thresholds. (a) Number of significant genes. (b) Fraction of total ESGs significant.

(a) Overlap between expanded gene sets

	Her2	Basal	LumA	LumB	Norm
Her2	458	4	61	88	8
Basal	4	509	0	0	103
LumA	61	0	519	86	13
LumB	88	0	86	552	0
Norm	8	103	13	0	499

(b) Overlap expressed as a %

	Her2	Basal	LumA	LumB	Norm
Her2	1.00	0.01	0.13	0.18	0.02
Basal	0.01	1.00	0.00	0.00	0.20
LumA	0.13	0.00	1.00	0.16	0.03
LumB	0.18	0.00	0.16	1.00	0.00
Norm	0.02	0.20	0.03	0.00	1.00

Supplementary Table 3: Overlap of extended signature genes (ESGs) between subtypes. (a) Number of overlapping genes. (b) Fraction of total ESGs per subtype that is overlapping.

**Luminal A**	
GATA3	10p15
TCF7L2	10q25
FOXM1	12p13
TEAD4	12p13
ELF1	13q13
FOXA1	14q12
MAX	14q23
TCF12	15q21
PML	15q22
SIN3A	15q22
NR2F2	15q26
JUND	19p13
CEBPB	20q13
ZNF217	20q13
FOSL2	2p23
HDAC2	6q21
ESR1	6q24
MYC	8q24
PGR	11q22
TBX3	12q24
ZBTB42	14q32
IRX5	16q11
SREBF1	17p11
CREB3L4	1q21
PBX1	1q23
XBP1	22q12
FOXP1	3p14
SPDEF	6p21
AR	Xq12

**Basal**	
CTBP2	10q26
NANOG	12p13
TEAD4	12p13
MAX	14q23
FOS	14q24
TCF12	15q21
STAT3	17q21
GTF2F1	19p13
JUND	19p13
BCL3	19q13
USF1	1q22
ATF3	1q32
RBBP5	1q32
CEBPB	20q13
BACH1	21q22
BCL11A	2p16
FOSL2	2p23
ATF2	2q32
REST	4q12
CHD1	5q15
EGR1	5q23
NR3C1	5q31
TAF7	5q31
SRF	6p21
POU5F1	6p21
HDAC2	6q21
TBP	6q27
MYC	8q24
RXRA	9q34
TAF1	xq13
ELF5	11p13
ETV6	12p13
SOX8	16p13
SOX9	17q23
RUNX3	1p36
ETS2	21q22
MAFF	22q12
SOX10	22q13
EN1	2q13
ETV5	3q28
ID4	6p22
FOXC1	6p25
FOXQ1	6p25
CREB3L2	7q34
NFIB	9p24
NFIL3	9q22

Supplementary Table 4: Subtype specific TFs derived from ChIP-seq and motif evidences

Luminal A motifs

TF	Motif	E-value
GATA3	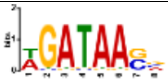	2.7e-52
PGR	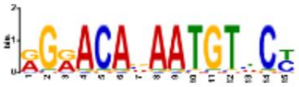	2.4e-4
TBX3	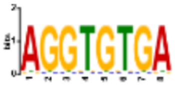	3.9e-5
FOXA1	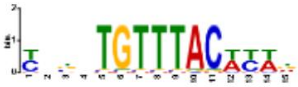	5.8e-197
IRX5	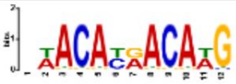	8.1e-4
SREBF1	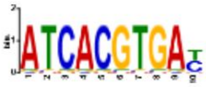	4.4e-4
CREB3L4	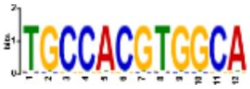	2.2e-37
PBX1	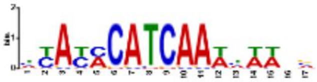	9.2e-1
XBP1	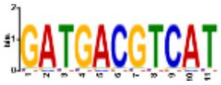	3.9e-4
FOXP1	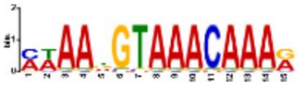	3.5e-60
SPDEF	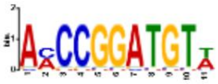	3.7e-17
ESR1	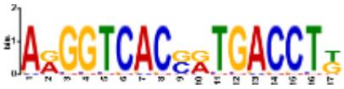	1.0e-30
AR	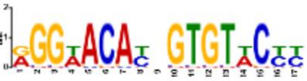	1.3e-3

Basal motifs

TF	Motif	E-value
ELF5	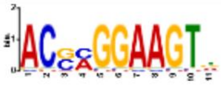	1.6e-18
ETV6	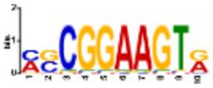	1.6e-18
SOX8	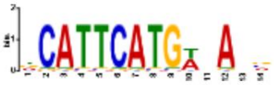	3.3e-3
SOX9	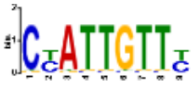	1.8e-2
RUNX3	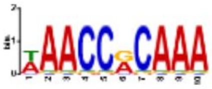	2.2e0
CEBPB	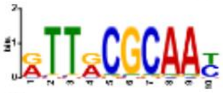	6.6e-27
ETS2	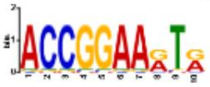	3.5e-12
MAFF	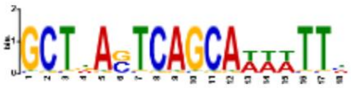	1.3e-34
SOX10	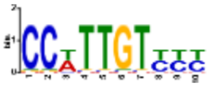	2.3e0
BCL11A	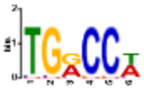	2.0e-2
EN1	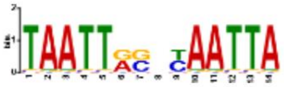	1.6e-25
ETV5	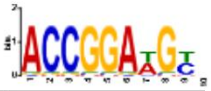	3.4e-13
ID4	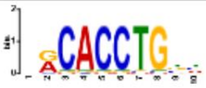	1.7e-11
FOXC1/FOXQ1	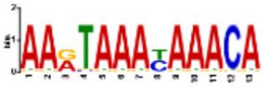	3.3e-8
CREB3L2	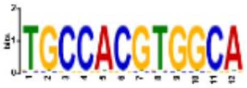	1.6e-12
NFIB	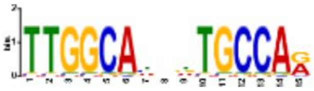	5.2e-5
NFIL3	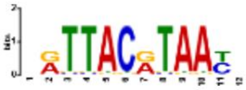	1.1e-15

Supplementary Table 5: Enrichment of motifs supporting motif-derived regulators

(a)

Query: ETS2 NFIL3 GRHL1 FOXQ1 ETV6 KLF5	Q-value	-log(Q-value)
blood vessel development	1.40E-03	2.85
blood vessel morphogenesis	2.21E-03	2.66
epidermis development	2.01E-02	1.70
response to stress	2.63E-03	2.58
angiogenesis	2.74E-03	2.56

(b)

Query: TCF7L2 NFIB ID4 BCL11A EN1	Q-value	-log(Q-value)
**stem cell differentiation**	8.95E-05	4.05
regulation of stem cell differentiation	3.40E-03	2.47
**cell adhesion**	4.88E-03	2.31
Wnt receptor signaling	1.30E-02	1.89

(c)

Query: ZNF532 ETV5 TCF7L1 ZNF521 IRX1	Q-value	-log(Q-value)
**stem cell differentiation**	1.25E-03	2.90
**extracellular matrix organization**	1.81E-09	8.74
**cell adhesion**	7.19E-08	7.14
**cell morphogenesis**	6.62E-07	6.18
cell migration	1.54E-03	2.81
**epithelial to mesenchymal transition**	3.68E-03	2.43
mesenchyme development	1.70E-03	2.77

(d)

Query: KLF11 RUNX3	Q-value	-log(Q-value)
cellular response to interferon gamma	3.70E-06	5.43
lymphocyte activation	1.09E-02	1.96
cytokine mediated signaling pathway	4.37E-04	3.36
regulation of defense response	1.97E-04	3.71
immune response activating signal transduction	2.84E-04	3.55

(e)

Query: FOXP1 ESR1 PBX1 FOXA1 MYB TSC22D3	Q-value	-log(Q-value)
response to estrogen stimulus	1.99E-02	1.70
carboxylic acid catabolic process	2.39E-03	2.62

Sources of Analysis Result

(a) http://seek.princeton.edu/viewer33.jsp?sessionID=1562601544876&sort_sample_by_expr=true
(b) http://seek.princeton.edu/viewer33.jsp?sessionID=1562601638317&sort_sample_by_expr=true
(c) http://seek.princeton.edu/viewer33.jsp?sessionID=1562601783245&sort_sample_by_expr=true
(d) http://seek.princeton.edu/viewer33.jsp?sessionID=1562601866390&sort_sample_by_expr=true
(e) http://seek.princeton.edu/viewer33.jsp?sessionID=1562604208028&sort_sample_by_expr=true
Select "Enrichment of genes" - "Analyze top genes: 200" - "GO Biological Process (BP) terms" - "Include Query: Yes"

Supplementary Table 6: GO-term enrichment of TF groups in the TF-TF regulatory network in Figure 2. GO-term enrichment was obtained by entering each TF-group as a query in SEEK, and next conducting a GO-analysis on the expanded gene-set.

Enhancer region comparison:
Overlap with the open chromatin ATAC-seq data on MDA-MB231. SRA ID: SRR7225842 (or GSM3161721) (R2)

ChIP-seq names (R1)	Cell line	TF	Overlap between ChIP-seq and SRR7225842 (R1)	Overlap between ChIP-seq and SRR7225842 (R2)	Total R1 peaks	Total R2 peaks	Fraction Overlap (R1)	Fraction Overlap (R2)
wgEncodeHaibTfbsA549Cebpbsc150V0422111	A549	Cebpb	**10732**	10566	32301	55186	0.332	**0.191**
wgEncodeHaibTfbsA549Fosl2V0422111Etoh02	A549	Fosl2	**15660**	15433	23660	55186	0.662	**0.280**
wgEncodeHaibTfbsA549Tcf12V0422111Etoh02	A549	Tcf12	**10789**	10451	14875	55186	0.725	**0.189**
wgEncodeHaibTfbsH1hescHdac2sc6296V0416102	H1hesc	Hdac2	8731	8240	20172	55186	0.433	0.149
wgEncodeHaibTfbsH1hescJundV0416102	H1hesc	Jund	6276	6118	10352	55186	0.606	0.111
wgEncodeHaibTfbsH1hescMaxV0422111	H1hesc	Max	**20358**	18761	57376	55186	0.355	**0.340**
wgEncodeHaibTfbsH1hescTead4sc101184V0422111	H1hesc	Tead4	**15609**	15037	44587	55186	0.350	**0.272**
wgEncodeHaibTfbsT47dEralphaaV0416102Est10nm1h	T47d	Eralphaa	2319	2299	11660	55186	0.199	0.042
wgEncodeHaibTfbsT47dFoxa1sc6553V0416102Dm002p1h	T47d	Foxa1	8535	8348	39407	55186	0.217	0.151
wgEncodeHaibTfbsT47dGata3sc268V0416102Dm002p1h	T47d	Gata3	6593	6497	29378	55186	0.224	0.118
wgEncodeHaibTfbsT47dP300V0416102Dm002p1h	T47d	P300	5600	5487	15702	55186	0.357	0.099
wgEncodeSydhTfbsH1hescCtbp2Ucd	H1hesc	Ctbp2	5809	5564	17630	55186	0.329	0.101
wgEncodeSydhTfbsMcf10aesCfosEtoh01Hvd	Mcf10aes	Cfos	**19396**	19123	53962	55186	0.359	**0.347**
wgEncodeSydhTfbsMcf10aesCmycTam14hHvd	Mcf10aes	Cmyc	**20276**	19705	40977	55186	0.495	**0.357**
wgEncodeSydhTfbsMcf10aesStat3Etoh01bStd	Mcf10aes	Stat3	**17176**	16856	46887	55186	0.366	**0.305**
combined.A549.Cebpb.Fosl2.Tcf12	A549	combined	**24355**	22025	52611	55186	0.463	**0.399**
combined.H1hesc.Hdac2.Max.Tead4	H1hesc	combined	**30096**	24288	93599	55186	0.322	**0.440**
combined.Mcf10aes.Stat3.Cfos.Cmyc	Mcf10aes	combined	**31085**	28831	84204	55186	0.369	**0.522**
combined.T47d.Eralphaa.Gata3.Foxa1	T47d	combined	13378	12463	59744	55186	0.224	0.226

Supplementary Table 7: Overlap between regulatory regions of A549, Mcf10aes, H1hesc ChIP-seq experiments and open chromatin ATAC-seq data of MDA-MB-231. Combined (last 4 rows) indicate pooled peaks of multiple ChIP-seqs.
