# Supplementary material for: Subtype-specific transcriptional regulators in breast tumors subjected to genetic and epigenetic alterations
Source: Bioinformatics. 2019 Sep 16;36(4):994–9. doi: 10.1093/bioinformatics/btz709 (PMC7031777; doi:10.1093/bioinformatics/btz709)
Supplement: btz709_Supplementary_Data [file btz709_supplementary_data.zip › Supplementary.Methods.pdf]

ChIP-seq derived TFs and motif-derived TFs.

We derived two categories of TFs relevant to cancer subtype-specific coexpressed genes. The first category is ChIP-seq-derived TFs. The relevant TFs are inferred by assessing if the list of ChIP'd binding sites in a given ENCODE experiment are significantly overlapping with the regulatory regions surrounding co-expressed genes found by SEEK. In details, we derived a binding score per gene (as outline above) for each ENCODE ChIP-seq experiment C_i. To determine if a ChIP-seq experiment C_i might be a regulator, the ranked list of genes (by binding score) in C_i is compared to coexpressed genes of subtype A for significance testing using minimal hypergeometric overlap statistic (GORILLA) (Eden *et al.*, 2009). GORILLA finds enrichment between a rank-list and the coexpressed genes without a need to specify depth of rank-list to compare with the gene-set. A relevant ChIP-seq experiment must satisfy $P < 1e\text{-}5$, and if so the ChIP'd TF becomes a relevant regulator. To perform the above analyses, TF ChIP-seq datasets were selected from cell lines MCF7, T47d for luminal A, and A549, H1-HESC, MCF10a-er-src for basal subtype. The cell lines A549, H1-HESC were chosen because there are no breast related mammary epithelial and stem cell lines in ENCODE with sufficient number of TF ChIP-seq datasets. On the other hand, based on the highly significant enrichment of binding near coexpressed genes (Fig 1), A549 and H1-HESC are each highly similar to the basal subtype (see Supplementary Fig 1).

The second category of TFs is inferred from the presence of binding motifs in the regulatory region of coexpressed genes ("motif-derived") with additional filtering applied. To find the motif-derived TFs in each subtype, we first get the regulatory sequences within 50kb of TSS for all coexpressed genes in a subtype. Regulatory sequences include TF binding sites from ChIP-seq experiments of cell lines that are relevant for each subtype (Supplementary Fig 1, red font). We then performed motif-enrichment analysis on these sequences using in vitro motif database JASPAR (Khan *et al.*, 2017) and Centrimo from MEME-ChIP suite (Machanick and Bailey, 2011) with default settings. Qualified motifs are then subject to a second stage of filtering, where we checked whether TFs corresponding to these motifs are coexpressed with the subtype seed genes. Only TFs of the motif family that are coexpressed with the subtype genes are named motif-derived regulators.

References:

Eden,E. *et al.* (2009) GOrilla: a tool for discovery and visualization of enriched GO terms in ranked gene lists. *BMC Bioinformatics*, **10**, 48.

Khan,A. *et al.* (2017) JASPAR 2018: update of the open-access database of transcription factor binding profiles and its web framework. *Nucleic Acids Res.*

Machanick,P. and Bailey,T.L. (2011) MEME-ChIP: Motif analysis of large DNA datasets. *Bioinformatics*, **27**, 1696–1697.
